# Changes in the growth and Lancemaside A content of *Codonopsis lanceolata* (deodeok) sprouts under LED-based lighting at different red/far-red ratios

**DOI:** 10.3389/fpls.2025.1548781

**Published:** 2025-05-28

**Authors:** Ye Lin Kim, Han-Sol Sim, Seong-Nam Jang, Jin-Hui Lee, Ki-Ho Son

**Affiliations:** ^1^ Department of GreenBio Science, Gyeongsang National University, Jinju, Republic of Korea; ^2^ Department of Practical Arts Education, Jeonju National University of Education, Jeonju, Republic of Korea; ^3^ Division of Horticultural Science, Gyeongsang National University, Jinju, Republic of Korea

**Keywords:** *Codonopsis lanceolata*, R/FR ratio, vertical farm, Lancemaside A, biomass, sprouts

## Abstract

**Introduction:**

*Codonopsis lanceolata* (deodeok) is used in traditional medicine because it contains saponins with high medicinal value. While previous studies have explored the general effects of red/far-red (R/FR) ratios on plant morphology and secondary metabolites, the specific impact of FR light on plant growth and bioactive compound accumulation of *C. lanceolata* sprouts remains largely unexplored.

**Methods:**

Here, we established a cultivation method for the *C. lanceolata* short-cycle sprout form on a vertical farm. Plants were grown under four different R/FR light conditions: white light (control), R/FR 3.0, R/FR 1.2, and R/FR 0.75. All treatments were provided with the same PPFD (210 ± 10 μmol m⁻² s⁻¹), and FR light was added to adjust the R/FR ratio. This setup was used to investigate the effects of varying R/FR ratios on plant growth and changes in bioactive compound accumulation.

**Results:**

FR supplementation significantly affected plant growth, development, and bioactive compound accumulation. Most growth parameters significantly increased as the R/FR ratio decreased. Adding FR light effectively increased the fresh and dry weight, plant height, leaf area, and node number. Moreover, the total phenolic content, flavonoid levels, and antioxidant capacity significantly increased at R/FR ratios of 1.2 and 0.75. The Lancemaside A content per plant was higher under FR supplementation than under white light treatment, slightly reducing at an R/FR ratio of 0.75 compared with that at 1.2, suggesting a possible inhibitory effect of excessive FR light.

**Conclusions:**

The findings indicate that appropriate FR light supplementation can enhance biomass and increase bioactive compounds. Thus, FR supplementation in a vertical farming system could boost the growth and bioactive substance content of sprouts, which has potential value for pharmaceutical and cosmetic applications.

## 1 Introduction

*Codonopsis lanceolata*, commonly known as deodeok, is a perennial vine that belongs to the Campanulaceae family. It is widely cultivated in East Asian regions such as Korea, China, and Japan because of its nutritional and medicinal value (Hwang et al., 2019). *C. lanceolata* is considered one of the five ginsengs and is known to be an effective medicinal plant for fever reduction, detoxification, and sore throat (Yoo et al., 2002). Lancemaside A, a triterpenoid saponin, is the primary active ingredient in *C. lanceolata* and its extracts (Kim et al., 2021). Along with its metabolites, Lancemaside A is frequently used as both a food and herbal medicine in Asian countries, including Korea, China, and Japan, and is applied for the treatment of inflammatory diseases, such as cough and bronchitis (Ushijima et al., 2008; Hyam et al., 2013). *C. lanceolata* is primarily cultivated in mountainous regions and open fields on general farms (Lee and Won, 2007), and its roots are mainly harvested in spring and fall (Lee et al., 2019). However, cultivating *C. lanceolata* requires a three-year period from sowing to harvest and a large cultivation area to ensure a stable supply (Kim et al., 2014). Additionally, because it must be grown in open fields, extensive management is necessary to control weeds, pests, and other environmental factors (Moon et al., 2018). As the value of *C. lanceolata* continues to increase and the challenges of open-field cultivation persist, interest in utilizing not only the roots but also other parts such as the leaves and stems for research purposes has increased (Kim et al., 2021). Although research on *C. lanceolata* is ongoing, additional studies on *C. lanceolata* sprouts, which can be cultivated for shorter periods, are required (Hwang et al., 2019).

Sprout vegetables germinate from seeds and develop into cotyledons (Kim et al., 2023), and they are used holistically during the early stages before total growth (Jun et al., 2012). Sprouts are considered a rich source of health-promoting natural products, such as amino acids, essential oils, polyphenols, minerals, and vitamins, and thus represent important food additives with diverse biological properties (Swieca et al., 2019). Sprouts are commonly used to enhance the taste and texture of salads (Kitazaki et al., 2015). Moreover, plants that present beneficial and medicinal properties have been used for a long time (Jain and Saklani, 1991). According to a World Health Organization report, the global medicinal plant market is expected to increase by 15–25% every year and reach $5 trillion by 2050 (Hishe et al., 2016). These sprouts can be classified into sprouts, microgreens, and baby leaves based on their developmental stage. Sprouts are generally grown under dark and high-humidity conditions for 4–10 days, with the entire plant, including the roots, typically consumed. Microgreens have a longer growth cycle (7–28 days) and refer to plants at the stage where true leaves have developed. Furthermore, the baby leaf stage refers to vegetable plants grown for 20–40 days, during which young leaves are primarily harvested and used (Di Gioia et al., 2017; Lenzi et al., 2019; Ebert, 2022). From this perspective, plants such as ginseng and deodeok, whose roots are traditionally used for medicinal purposes, have different growth forms and utilization compared to sprouts of leafy vegetables. Therefore, the term “sprout” can be applied to medicinal crops like ginseng, which are consumed with their roots. Previous studies have also classified ginseng plants grown for 3–6 weeks as ginseng sprouts, which have garnered significant attention due to their higher saponin content in the leaves compared to the roots, despite their short cultivation period (Kim et al., 2020). Additionally, their shorter growth cycle and high nutritional value have contributed to the widespread use of ginseng sprouts as raw materials for processed products (Seong et al., 2019).

A vertical farm is a multilevel indoor system with artificial lighting in which all environmental conditions are independently controlled relative to the external environment (Carotti et al., 2023). This approach to food production is gaining attention because of its ability to increase crop yield and uniformity, enable aseptic production, and reduce cultivation periods (Heo et al., 2013; Dou et al., 2020). Vertical farming has also been highlighted as a strategy for enhancing resource use efficiency, making it a sustainable option for large-scale food production (Orsini et al., 2020). In these closed cultivation systems, where environmental parameters are meticulously controlled, light quality is one of the most critical factors influencing plant growth and development (Kotiranta et al., 2024). To optimize indoor plant growth, efforts have been made to manipulate the spectral composition of light to closely mimic natural sunlight, which represents an effective strategy for enhancing growth and nutrient contents (Mobini et al., 2016).

In vertical farming systems, wherein environmental conditions are precisely controlled, light quality is a crucial factor for optimizing plant growth and development. Plants respond to light through a range of metabolic and morphogenetic changes that are heavily influenced by the quality and quantity of the light they receive. A critical factor in this light response is the ratio of red (R) to far-red (FR) light (R/FR), which plays a key role in regulating plant growth and development (Pashkovskiy et al., 2024). Plant leaves typically absorb light efficiently in the blue and R regions of the spectrum (R, 600–700 nm) but reflect most light in the green and FR regions (FR, 700–800 nm). In addition, leaves exhibit high transmittance in the FR region (Tegelberg et al., 2004). Under natural sunlight, the R/FR ratio is approximately 1.2, although this ratio significantly decreases to 0.1–0.7 in shaded environments and can increase to approximately 7 under LED illumination (Smith, 1982). A low R/FR ratio is commonly associated with shade-avoidance responses, which include reduced branching, elongation of stems, petioles, and leaves, and increased apical dominance and shoot dry weight (Fernández-Milmanda and Ballaré, 2021). These adaptive changes enable plants to compete for light in crowded environments.

Recent studies have highlighted the importance of manipulating the R/FR ratio to optimize plant growth. For instance, in the model plant *Arabidopsis thaliana*, a decrease in the R/FR ratio due to increased FR light leads to enhanced stem elongation (Finlayson et al., 2010; Sasidharan et al., 2010). Another study showed that varying the R/FR ratio improved the growth and total phenol content of the medicinal plant *Crepidiastrum denticulatum* (Bae et al., 2017). Similarly, research conducted in controlled plant factory settings demonstrated that increasing the R/FR ratio can significantly boost the growth and production of bioactive compounds in lettuce (Lee et al., 2015, 2016). These findings underscore the critical role of the R/FR ratio in regulating growth and biomass accumulation, suggesting that optimizing this ratio can have significant implications for both agricultural productivity and plant-derived product quality (Ammer, 2003). To the best of our knowledge, studies investigating the effects of light quality on *C. lanceolata* have been limited to analyzing the impact of white, red, green, and blue light combinations on seedling quality (Liu et al., 2019; Hwang et al., 2023) and examining growth characteristics under far-red light as the sole light source (Ren et al., 2018). However, changes in the morphological and physiological characteristics induced by varying R/FR ratios have not been reported.

Although the effects of R/FR ratios on plant growth and secondary metabolism in various crops have been well-documented, their specific influence on plant growth and bioactive compound accumulation in *C. lanceolata* sprouts is poorly understood. Therefore, we aimed to establish a large-scale production system for *C. lanceolata* sprouts using a controlled vertical farming environment. Moreover, the antioxidant properties and secondary metabolite contents of *C. lanceolata* sprouts were investigated under varying R/FR light ratios. This research seeks to provide essential data on the cultivation of *C. lanceolata* sprouts as a functional health food and valuable medicinal crop.

## 2 Materials and methods

### 2.1 Plant materials

The *C. lanceolata* seeds used in this study were obtained from Dong Won Nongsan Seeds Co., Ltd. (Yongin, South Korea). Before sowing, the seeds were soaked in a gibberellin aqueous solution (IAP, Jahng Ryu Industries Co., Ltd., Cheongju, South Korea) for 1 day, soaked in 70% ethanol for 30 s, and rinsed three times with distilled water. Subsequently, they were sterilized with 20% NaOCl (Duksan Science Co., Ltd., Seoul, South Korea) for 15 min and rinsed four times with distilled water.

Gibberellin treatment has been applied to enhance the efficiency of germination in C. *lanceolata*, which exhibits a naturally low germination rate due to the dormancy imposed by the seed coat (Ghimire et al., 2006). Previous research has indicated that, even after gibberellin treatment, C. *lanceolata* seeds require a considerable duration to produce leaves once all of the seeds have germinated. Consequently, sprout growth persists for several weeks until it attains a stage at which experimental treatment can be administered.

### 2.2 Cultivation conditions

*C. lanceolata* seeds (3 g) were sown into a tray (31.5 × 23 × 7.5 cm, L × W × H) with drain holes filled with a commercial ginseng soil mix medium (Myeongpum-Insamsangto, Shinsung Mineral Co., Ltd., Goesan, South Korea) in a cultivation room. After sowing, germination proceeded in the dark for 7 days and then under light conditions. The environmental conditions of the cultivation room were as follows: temperature of 23 ± 2°C, relative humidity of 50 ± 10%, and 210 ± 10 µmol m^-2^ s^-1^ [photosynthetic photon flux density (PPFD); white LEDs, 12 h photoperiod]. Additionally, to focus solely on light quality differences, the light source was configured such that the PPFD in the 400–700 nm wavelength range was maintained at 210 ± 10 µmol m^-2^ s^-1^ when FR light was added. The PPFD levels were measured using a quantum sensor (Li-180; Li-Cor, Lincoln, NE, USA), and temperature and humidity were monitored and regulated daily using a wireless data logger (TH2; Efento, Krakow, Poland). The plants were irrigated using Hoagland’s nutrient solution (pH, 6.5; electrical conductivity [EC], 1.5 dS m^-1^). Irrigation was performed using the bottom irrigation method.

The experiments were conducted on seedlings that had fully expanded true leaves, rather than at the early cotyledon stage, with the objective of ensuring uniform plant growth. This decision was informed by earlier research demonstrating that C. *lanceolata* requires a prolonged period to establish sprouts and that the impact of light quality on biomass accumulation and the synthesis of secondary metabolites may not be completely reflected in early growth stages (Ghimire et al., 2006; Hwang et al., 2019).

### 2.3 Light treatment

**Figure 1:**
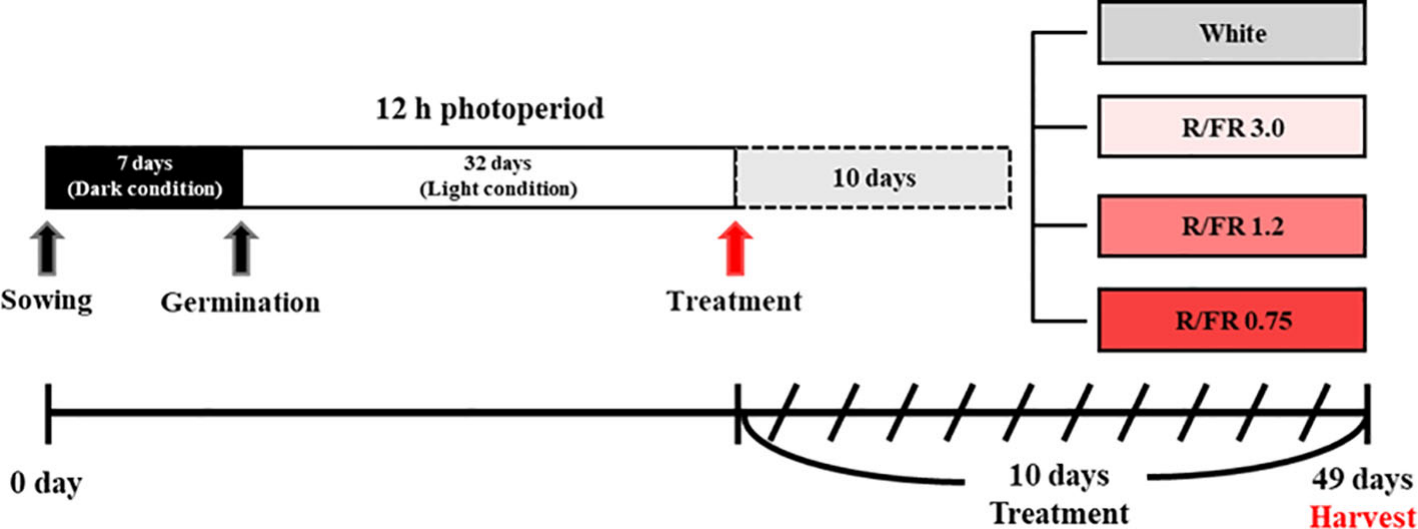
Schematic diagram of the experimental lighting conditions according to the red/far-red (R/FR) ratio.

**Figure 2:**
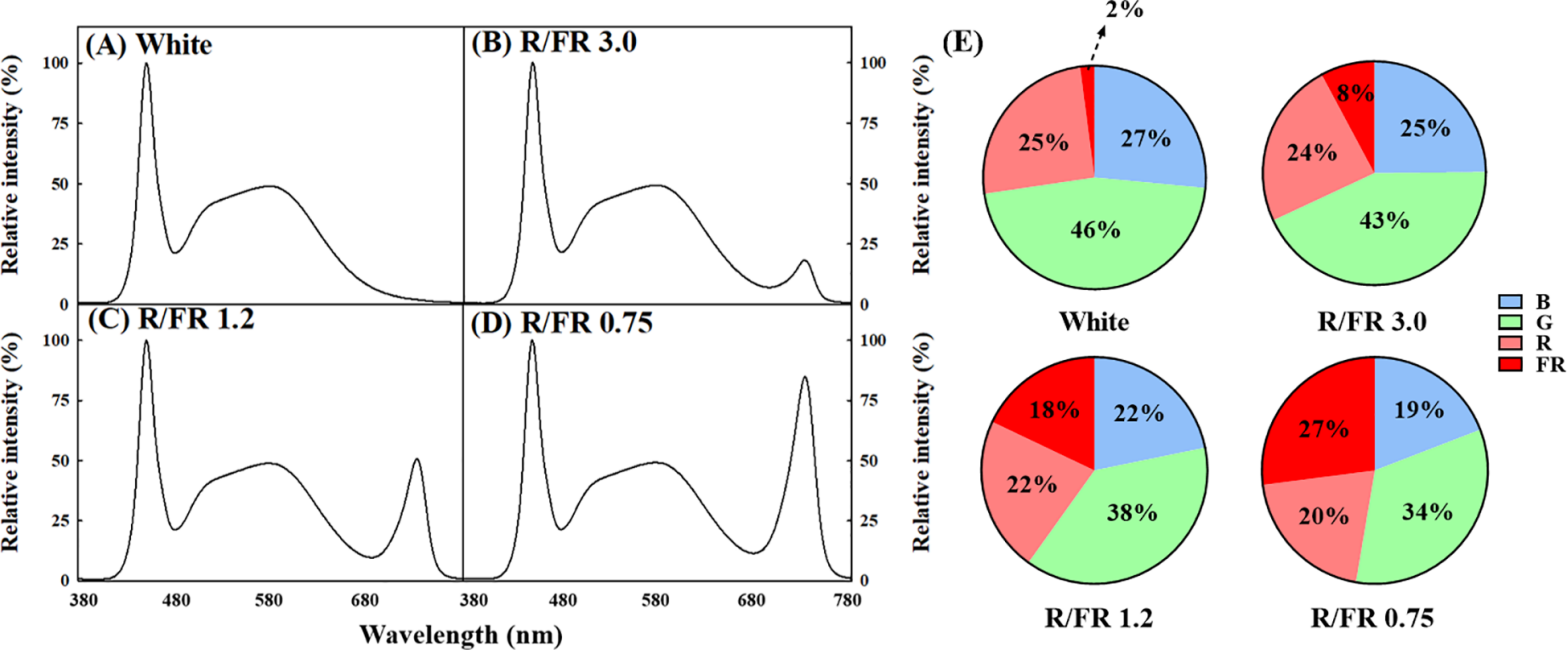
Relative spectral distribution according to the four red/far-red (R/FR) ratio treatments. White **(A)**, R/FR ratio 3.0 **(B)**, R/FR ratio 1.2 **(C)** and R/FR ratio 0.75 **(D)**. The different colors represent different light qualities in figures **(E)**. The numbers in panel **(E)** represent the percentage of relative spectral intensity.

A separate plant growth bed in which the ratio of R to FR light could be adjusted was manufactured and used in the experiment for the cultivation of *C. lanceolata* sprouts. The effects of four light quality treatments, including the control, were verified on the sprouts of *C. lanceolata* 39 days after sowing the seeds. Supplemental light treatment was performed for 10 days (**Figure 1**). The R/FR ratio treatment added FR to white light to adjust the R/FR ratio. For FR control, a Samsung LED (LH351H Far-Red 730 nm, Samsung Electronics, Suwon, South Korea) was used. The treatments included R/FR values of 3.0 (**Figure 2B**), 1.2 (**Figure 2C**), and 0.75 (**Figure 2D**) and white light only (**Figure 2A**). The relative spectral distribution for each treatment, including the proportions of red, far-red, blue, and green light, is illustrated in **Figure 2E**. The light spectrum distribution was measured using a portable spectroradiometer (Li-180, Li-Cor, Lincoln, NE, USA) and divided into six zones for each treatment group (**Table 1**).

**Table 1:** Average photon flux (µmol m^-2^ s ^-1^) of the wavelength according to the light source.

Treatment	PPFD 400–700 nm (µmol m^-2^ s^-1^)	PFD 380–780 nm (µmol m^-2^ s^-1^)	Spectrum				R/FR ratio
			B (%)^z^	G (%)	R (%)	FR (%)	
White	212.87 ab	217.59 c	26.46	46.13	25.23	2.08	12.15
R/FR 3.0	206.13 b	223.86 c	24.81	43.21	24.06	7.83	3.07
R/FR 1.2	212.61 ab	259.17 b	21.83	38.12	22.09	17.88	1.23
R/FR 0.75	214.62 a	294.17 a	19.17	33.53	20.25	26.96	0.75

z Mean percentage of photon flux density (PFD) (B, blue; G, green; R, red; FR, far-red). Different letters indicate significant differences within columns at *p<* 0.05.

### 2.4 Growth characteristics and physiological measurements

The fresh weight (FW), dry weight (DW), dry matter content (DMC) (Kim and Hwang, 2019), node number, plant height, leaf area, and Fv/Fm were measured 49 days after sowing to compare the growth of *C. lanceolata* according to the R/FR ratio. A digital scale (PAG214C; Ohaus Corp., Parsippany, New Jersey, USA) was used to measure the FW and DW. A leaf area measuring instrument (Li-3100, LICOR, Lincoln, Nebraska, USA) was used to measure the leaf area. Chlorophyll fluorescence was measured by selecting the first largest leaves of *C. lanceolata*’s aerial parts. Five plants from each treatment group were selected as biological replicates. After dark adaptation for 15 min, the Fv/Fm values of true leaves were measured using a portable chlorophyll fluorometer (OS30P +, OPTI-SCIENCES, USA). Measurements were performed on the day of harvest. Samples were dried in an oven dryer (EP-20, KOTES, Co., Ltd., Seoul, South Korea) at 55°C for 72 h for DW determination.

The dry matter content was calculated using the following formula:


$$\begin{array}{c} \text{Dry matter content }\left(\%\right)\\ =\left(\text{dry weight of shoot }\left(\text{g}\right)/\text{fresh weight of shoot }\left(\text{g}\right)\right)\;\times \;100 \end{array}$$

To assess the growth characteristics and plant productivity, the compactness and leaf area ratio (LAR) (Moon et al., 2023) were computed using the following equations:


Compactness=shoot dry weight (g)/plant height (cm)

$$\text{LAR}=\text{leaf area }\left({\text{cm}}^{2}\right)/\text{shoot dry weight (g)}$$

### 2.5 Light and energy use assessment

The daily light integral (DLI) necessary for calculating the light use efficiency (LUE) was computed based on the photon flux density (PFD) to examine the specific effects of far-red light on plant productivity. LUE was derived as the ratio of biomass (fresh and dry weights) to the total mol of light (PFD) received, thus isolating the contribution of far-red light. For energy use efficiency (EUE), only FR supplemental lighting consumption at a set consumption rate of 60 W was considered because the white control did not use any FR light. Lighting was operated for 12 h per day over a 10-day period. LUE and EUE (Carotti et al., 2024) were calculated using the following formulas:


LUE=shoot fresh and dry weight (g)/DLI(mol),

$$\begin{array}{c} \text{EUE}=\text{shoot fresh and dry weight }\left(\text{g}\right)/\text{energy used }\left(\text{kWh}\right)\\ \times {\text{PFD}}_{\text{FR}} \end{array}$$

### 2.6 Analysis of bioactive compounds and antioxidant properties

The phenolic content was determined using a modified version of the Folin-Denis method (Ainsworth and Gillespie, 2007). Dry powdered samples (0.015 g) were combined with 80% acetone (1.5 mL) and sonicated for 15 min. The mixture was then stored at 4°C for 12 h. After centrifugation for 2 min at 13,000 rpm (WiseSpin CF-10; Daihan, Korea), the resulting supernatant was collected for analysis. In a 2-mL microcentrifuge tube, 0.135 mL of distilled water, 0.75 mL of 10% Folin-Ciocalteu reagent, 0.05 mL of the sample extract, and 0.6 mL of 7.5% Na_2_CO_3_ were sequentially added and mixed. A blank was prepared by substituting 0.05 mL of 80% acetone for the sample extract. The samples were vortexed for 10 s and then placed in a water bath at 45°C for 15 min. After cooling, absorbance was measured at 765 nm (Libra S32, Biochrom, France). The total phenolic content of *C. lanceolata* was expressed as milligrams of gallic acid equivalent (mg GAE) per plant (mg GAE/plant).

To measure the total flavonoid content, the method described by Pękal and Pyrznska (Pękal and Pyrzynska, 2014) was employed with slight modifications. Flavonoids were extracted from 50 mg of dried *C. lanceolata* plant material using 1 mL of 70% ethanol, followed by sonication for 15 min. The resulting extract was centrifuged at 13,000 rpm for 2 min (WiseSpin, CF-10; Daihan, South Korea), and the supernatant was collected for analysis. In a 1.5 mL microcentrifuge tube, 750 µL of distilled water, 45 µL of 5% NaNO_2_, and 150 µL of the sample extract were combined and allowed to react for 6 min. Next, 90 µL of 10% AlCl_3_ was added, and the mixture was incubated for 5 min. Further, 300 µL of 1 M NaOH and 165 µL of distilled water were added, followed by vortexing. A blank sample was prepared using 150 µL of 70% ethanol instead of the sample extract. The absorbance of each sample was recorded at 510 nm (Libra S32; Biochrom, France), and the total flavonoid content was expressed as milligrams of (+)-catechin per plant (mg (+)-catechin/plant).

The antioxidant capacity was assessed using the ABTS radical cation decolorization method, which measures the scavenging activity of 2,2’-azino-bis(3-ethylbenzothiazoline-6-sulfonic acid) (Miller and Rice-Evans, 1996). For this, 15 mg of the dry powder sample was combined with 1.5 mL of 80% acetone and sonicated for 15 min, followed by storage at 4°C for 12 h. After centrifugation for 2 min, the supernatant was collected and analyzed. Absorbance was measured at 730 nm (Libra S32, Biochrom, France). The antioxidant capacity of *C. lanceolata* is expressed as mM Trolox equivalents per plant (mM Trolox/plant).

### 2.7 Liquid chromatography-mass spectrometry analyses of Lancemaside A

Dried powdered samples (5 g) were extracted with 20 mL of 70% methanol (HPLC-grade) in a constant temperature bath at 70°C for 1 h. The mixture was then centrifuged at 3,000 rpm for 10 min (1730R, GYROZEN Co., Ltd., Gimpo, South Korea), and the supernatant was filtered through a 0.45 µm membrane filter. This extraction process was repeated twice to obtain 40 mL of *C. lanceolata* sprout extract. The collected extracts were concentrated under reduced pressure at 60°C, dissolved in HPLC-grade water, and filtered through a syringe filter prior to analysis (Jang et al., 2022).

The extract was injected into an LIChrospher^®^100 RP-18 5-μm column (Merck Eurolab, Darmstadt, Germany) connected to a Shimadzu Nexera X2 UHPLC system (Shimadzu, Kyoto, Japan). The liquid chromatography system was coupled to a SCIEX QTRAP 4500 mass spectrometer with a Turbo V Ion Source and Turbo Ion Spray probe for electrospray ionization (SCIEX, Framingham, MA, USA). The mobile phases consisted of solvent A (water with 0.1% formic acid) and solvent B (acetonitrile with 0.1% formic acid), and a gradient elution program was used. The gradient profile was changed from 10% A at 4 min to 90% B at 6 min at a flow rate of 0.4 mL/min. The MS/MS conditions were as follows: electrospray ionization (ESI) source ion spray voltage of −4500 V (negative ion mode), source temperature of 500°C, and ion source gas pressures of 50 psi (GS1), 60 psi (GS2), and 25 psi (curtain gas, CUR). The Lancemaside A content was quantified based on a standard curve and expressed as milligrams per plant (mg/plant).

### 2.8 Correlation analysis

Pearson correlation coefficients were calculated using the mean values of 12 parameters, including growth traits, physiological indices and bioactive metabolite contents. A heat map was generated using GraphPad Prism (GraphPad Software, San Diego, CA, USA). Significant correlations were indicated at *p* < 0.05, *p* < 0.01 and *p* < 0.001.

### 2.9 Statistical analysis

The relationships between changes in growth parameters and secondary metabolites for each plant were determined via regression analysis based on the light spectrum ratios (%) of various blue, green, red, and FR light. Statistical analyses and graph plotting were performed using GraphPad Prism. Data are expressed as the mean ± standard deviation (SD). One-way analysis of variance (ANOVA) followed by Tukey’s *post-hoc* test was used to compare the means between groups. Statistical significance was set at *p*< 0.05.

## 3 Results and discussion

### 3.1 Growth characteristics under the R/FR treatments

**Figure 3:**
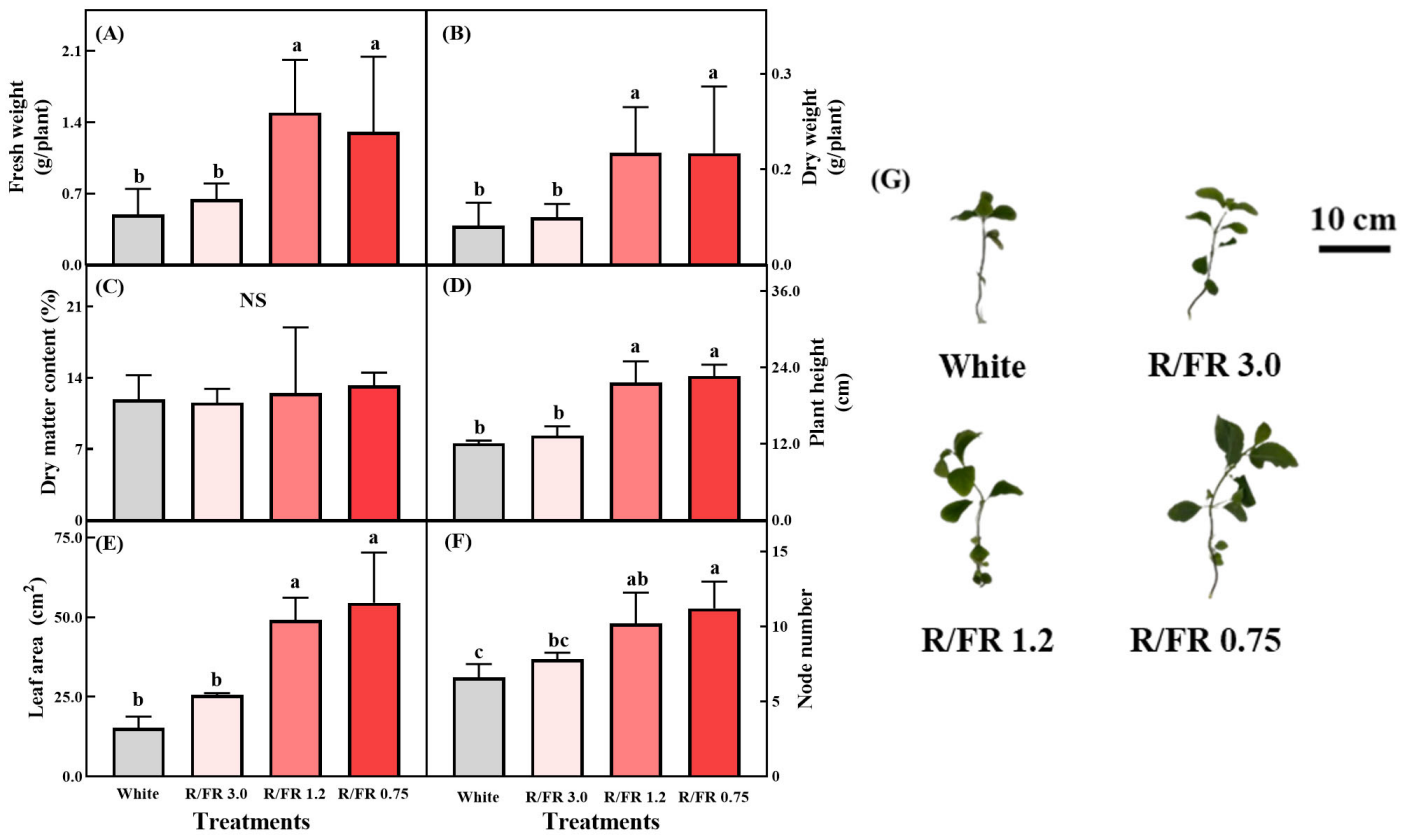
Fresh weight **(A)**, dry weight **(B)**, dry matter content **(C)**, plant height **(D)**, leaf area **(E)**, and node number **(F)** under the different red/far-red (R/FR) ratios. Growth of *C. lanceolata* sprouts under the different R/FR ratio treatment **(G)**. The error bars represent the standard deviations of 10 repetitions **(A–C)** or 5 repetitions **(D–F)**. Different lowercase letters on the bar chart indicated significant differences among treatments according to Tukey’s test (*p<* 0.05). *NS*, not significant.

The growth of *C. lanceolata* varied under the different R/FR ratios (**Figure 3G**). The FW and DW increased significantly in the R/FR 1.2 and 0.75 treatments (**Figures 3A, B**). However, the dry matter content did not show significant differences under the R/FR treatments (**Figure 3C**). Plant height (**Figure 3D**) and leaf area (**Figure 3E**) increased in the R/FR 1.2 and 0.75 treatments. The node number was significantly higher in the R/FR 1.2 and 0.75 treatments than in the white light control (**Figure 3F**).

Plants must obtain light energy for survival and adapt their growth and development to changes in light quality and intensity (Yuan et al., 2017). In particular, the R/FR ratio regulates growth processes throughout the plant life cycle (Demotes-Mainard et al., 2016). Under sunlight, the R/FR ratio is lower at the beginning and end of the photoperiod (approximately 0.6) than at solar noon (approximately 1.0–1.3) (Smith and Holmes, 1977). In the present study, as the R/FR ratio decreased, the FW, DW, plant height, and node number increased. Reducing the R/FR ratio improves petiole length by stimulating the activity of xyloglucan endotransglucosylase/hydrolase (XTH), an essential protein involved in cell-wall loosening (Sasidharan et al., 2010). DMC is closely linked to the leaf water content, which affects the cell protoplasm volume (Garnier and Laurent, 1994). It is also a reliable indicator of the plant’s response to nutrient stress (Gross et al., 2007). Recent studies have shown that precise regulation of the far-red light ratio can enhance yield and water use efficiency without negatively affecting DMC (Carotti et al., 2023). In the present study, no significant differences were observed in DMC among the control and the three light treatments, indicating that the different light spectra did not alter the balance between dry and fresh biomass in *C. lanceolata* sprouts. This consistency suggests that while the light treatments may have affected other growth characteristics, such as total biomass and plant height, the plant was able to maintain a stable DMC across all treatments.

In addition, a low R/FR-induced shade-avoidance strategy leads to expanded growth to capture more light (Runkle and Heins, 2001). Leaf area was significantly higher in both the R/FR 1.2 and 0.75 treatments, indicating that the plants responded similarly to these R/FR ratios. The increase in plant height observed under lower R/FR conditions may result from both leaf elongation and changes in leaf angle. Far-red light induces hyponasty, where leaves grow more upright, enhancing light interception by increasing canopy area (Liu and Van Iersel, 2023). Node number and plant height were highest in the R/FR 0.75 treatment. The observed increase in dry weight in the R/FR 1.2 and 0.75 treatments was attributed to the expansion of the leaf area. This is consistent with previous findings showing that increased far-red light exposure is associated with increased leaf area and greater biomass accumulation (Li and Kubota, 2009). However, because far-red photons are less efficiently absorbed than red photons, an excessive shift toward far-red light may reduce parabolic aluminized reflector (PAR) absorption and potentially lower photosynthetic efficiency (Liu and Van Iersel, 2023).

**Figure 4:**
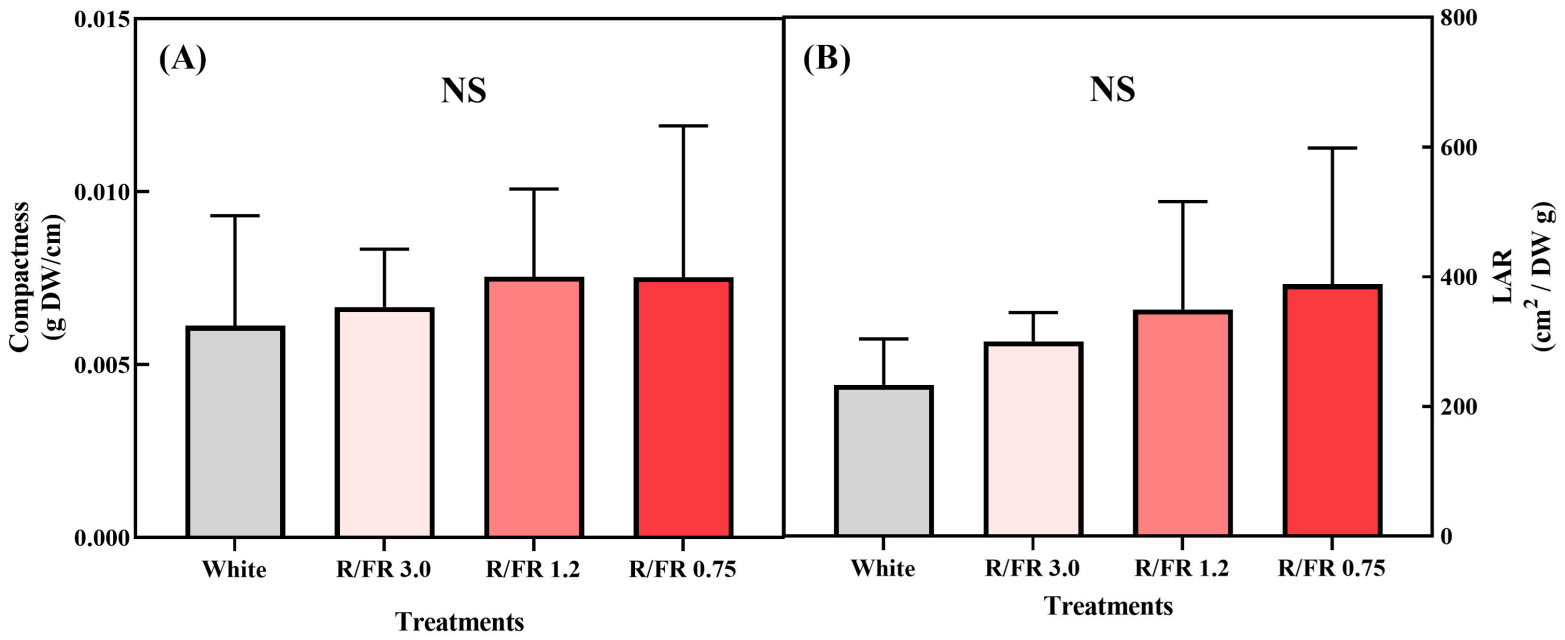
Effects of the red/far-red (R/FR) ratios on the compactness and leaf area ratio (LAR) in *C. lanceolata* sprouts **(A, B)**. *NS*, not significant (n=5).

The effects of different R/FR ratios on the compactness and LAR of *C. lanceolata* sprouts are presented in **Figure 4**. Significant differences were not observed in compactness (**Figure 4A**) or LAR among the treatments (**Figure 4B**).

Higher compactness values signify shorter and denser seedlings (Jeong et al., 2020). It is generally understood that far-red supplementation reduces compactness by producing thinner leaves (Demotes-Mainard et al., 2016). However, in this study, no significant differences were observed in compactness, even under the far-red treatments. This suggests that plant responses, including growth traits, such as plant length, may vary significantly across species, with some showing positive effects and others showing little to no response (Hwang et al., 2020).

During the treatment period, the DW and leaf area increased, although the LAR values did not show significant differences under the far-red treatment, despite a slight numerical increase. Generally, LAR tended to be higher under the high R/FR treatment than the low R/FR treatment because leaf allocation increases with higher R/FR (Cowan and Reekie, 2008). However, the lack of significance in this study suggests that FR treatment did not strongly influence the allocation toward leaf expansion compared with dry matter production. This finding is consistent with the results in cucumbers, where both leaf area and biomass increased under the FR treatment (Kusuma and Bugbee, 2023).

Such stable LAR values imply that *C. lanceolata* may have prioritized biomass accumulation over leaf expansion as FR exposure increased. Another consideration is that FR light may enhance biomass accumulation by increasing photosynthetic capacity without necessarily promoting proportional leaf area expansion, leading to stable LAR values (Shibuya et al., 2016; Meng and Runkle, 2019). This suggests that under the FR treatment, *C. lanceolata* may have allocated more resources toward dry matter accumulation than leaf area expansion, resulting in a non-significant LAR change despite the slight numerical increases.

### 3.2 Photosynthetic efficiency and chlorophyll content analysis based on the Fv/Fm and SPAD values under the R/FR ratio treatment

**Figure 5:**
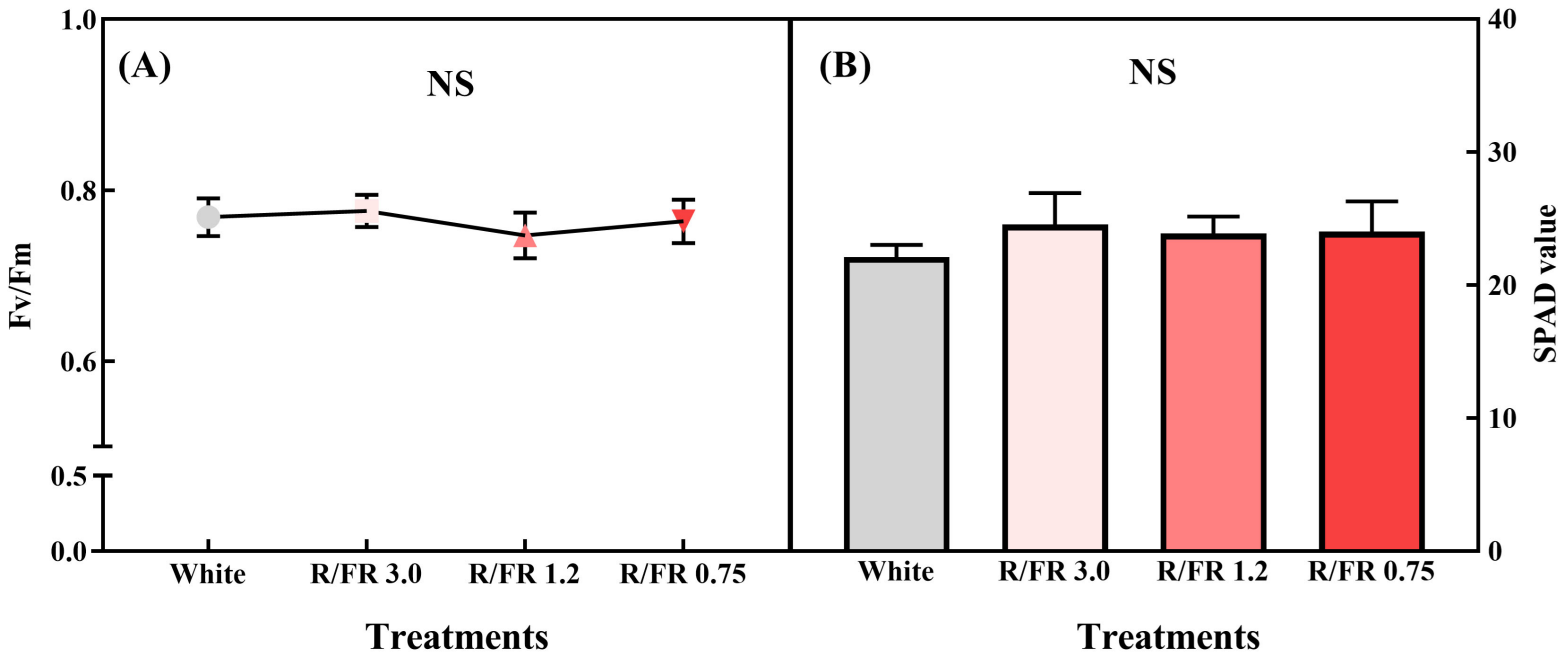
Fv/Fm **(A)** and SPAD value **(B)** of *C*. *lanceolata* sprouts under the different red/far-red (R/FR) ratios. The vertical error bars represent the standard deviations (n = 5). *NS*, not significant.

Significant differences were not observed in the maximum quantum efficiency of photosystem II (Fv/Fm) and SPAD values between the white control and treatment groups (**Figures 5A, B**). This finding reveals that the quantum potential of photosynthetic efficiency in plants can be assessed under different light conditions (Gonçalves and Santos, 2005). Healthy plants typically exhibit an Fv/Fm value of approximately 0.83, which reflects optimal photosynthetic efficiency (Lichtenthaler, 1988; Palliotti et al., 2005). In this study, the Fv/Fm values were maintained between 0.75 and 0.8 across all treatments, indicating that the FR treatment did not induce significant light stress, despite the slightly lower values than the optimal range reported in other studies (Mengarda et al., 2009). Since Fv/Fm is closely related to PPFD, plants can respond differently depending on the PPFD level. Therefore, monitoring such indicators is essential to ensure appropriate light use without imposing stress on plant growth (Chen et al., 2022).

The consistency of the Fv/Fm values in the FR treatments aligns with previous reports on tomatoes, which found no significant changes in photosynthetic efficiency under similar light conditions (Zhou et al., 2021). Moreover, SPAD measurements, which quantify the chlorophyll content and are often used to assess subtle changes in plant health, did not significantly differ between the control and FR treatments. This finding aligns with previous results on kale, where FR supplementation did not significantly affect SPAD values, suggesting that FR irradiation did not alter the chlorophyll content (Spencer et al., 2020).

### 3.3 Bioactive compounds, antioxidant properties, and Lancemaside A contents through R/FR ratio treatment

**Figure 6:**
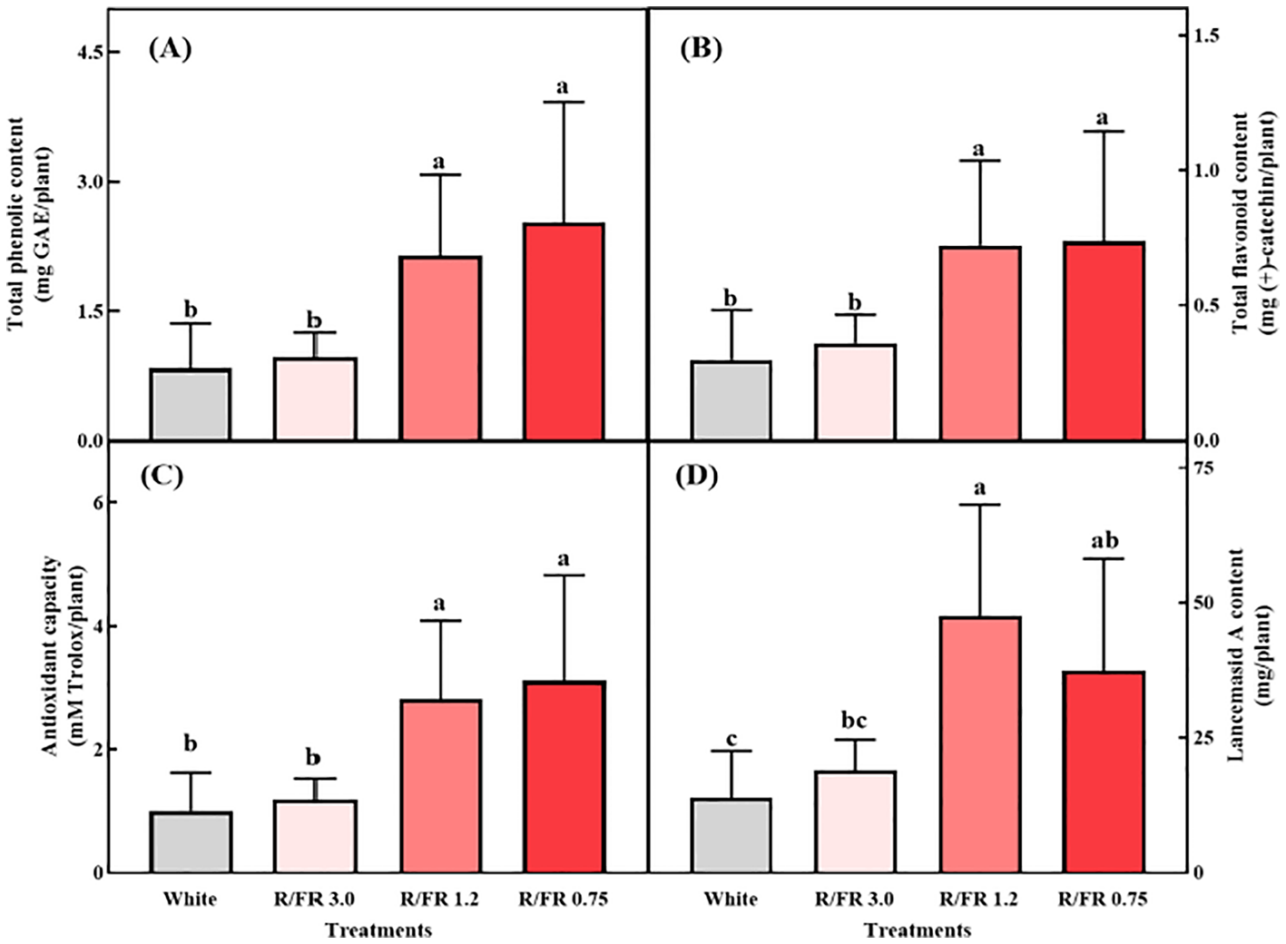
Effects of the different R/FR ratio treatments on *C. lanceolata* sprouts. Total phenolic content **(A)**, flavonoid content **(B)**, antioxidant capacity **(C)**, and Lancemaside A content **(D)**. The vertical bars represent the standard deviations (n = 9). *NS*, not significant.

The total phenolic (**Figure 6A**) and total flavonoid contents (**Figure 6B**), antioxidant capacity (**Figure 6C**), and Lancemaside A content (**Figure 6D**) per plant were all significantly higher in the R/FR 1.2 and 0.75 treatments. The total phenols, total flavonoids, and antioxidant capacity were analyzed to observe changes in secondary metabolites according to the R/FR ratio.

The concentrations of plant phytochemicals, such as phenolics and flavonoids, are significantly influenced by different light quality treatments (Li and Kubota, 2009). Although previous studies have suggested that higher FR light exposure can reduce the concentrations of phenolics and flavonoids in plants (Li and Kubota, 2009), the present study revealed that increased FW and DW in the R/FR treatments led to higher total amounts of these secondary metabolites per plant. This may be because plants allocate resources toward expansive growth rather than directly for phytochemical synthesis (Shibuya et al., 2012; Meng et al., 2019). This suggests that despite potential decreases in concentration per gram, the overall yield of phenolics, flavonoids, and antioxidant capacity per plant can increase due to enhanced biomass. This finding aligns with the results for *C. denticulatum*, where supplemental FR light significantly enhanced the growth and total phenolic content per plant (Bae et al., 2017), thus demonstrating that the phenolic content is directly linked to FR-induced plant growth.

Light is a key environmental factor that influences plant antioxidant activity. Studies on radish, Chinese cabbage, and broccoli have shown that supplemental far-red light enhances the antioxidant capacity (Jo and Lee, 2018). In particular, previous studies reported that a higher proportion of FR light at an R/FR ratio of 0.6 increased antioxidant capacity, likely due to elevated antioxidant enzyme activity under lower R/FR conditions (Wang et al., 2021). Plants may perceive FR light treatment as a signal of shaded conditions and potential competition, leading to increased antioxidant activity (Pashkovskiy et al., 2024). These results underscore the potential of optimizing *C. lanceolata* sprout cultivation with FR light supplementation to enhance biomass and bioactive compound production in a controlled plant production system.

The Lancemaside A content per plant was significantly higher in the R/FR 1.2 and 0.75 treatments, with the highest content per plant in the R/FR 1.2 treatment group (**Figure 6D**). The Lancemaside A content per plant was approximately 3.4, 2.67, and 0.7 times higher in the R/FR 1.2, 0.75, and 3.0 treatments, respectively, than in the white treatment group.

In conclusion, FR light supplementation effectively increased the content of Lancemaside A, a key secondary metabolite of *C. lanceolata*. Although excessive levels of FR light (R/FR 0.75) slightly decreased the Lancemaside A content (**Figure 6D**), a significant increase at lower R/FR ratios of 1.2 and 0.75 was still observed compared with that in white light (control). In conclusion, although Lancemaside A showed a slight numerical decrease at an R/FR of 0.75, this reduction was not statistically significant. Lancemaside A is a triterpenoid saponin and an essential secondary metabolite known for its anti-inflammatory effects (Lee et al., 2019). Traditionally, *C. lanceolata* has been used as an herbal medicine to treat conditions such as insomnia and other inflammatory diseases (Jung et al., 2012). Although previous studies have not determined the effects of FR light on Lancemaside A content, saponin levels have been shown to fluctuate in response to various abiotic factors (Szakiel et al., 2011). In this study, FR supplementation led to significantly higher Lancemaside A contents in the R/FR 1.2 and 0.75 treatments than in the white control group. These results are consistent with findings in ginseng sprouts, where FR light treatment increased the saponin and ginsenoside content (Kim et al., 2020).

The slight decrease in Lancemaside A content observed at an R/FR ratio of 0.75 suggests that excess FR light may influence metabolic pathways, potentially through its effect on jasmonic acid (JA) signaling (Kegge et al., 2013). JA is crucial for the regulation of secondary metabolite synthesis, and previous studies have shown that excessive FR light can suppress JA-responsive pathways (Courbier et al., 2021). Supplemental FR light reduces JA responses, thereby inhibiting the accumulation of metabolites, such as phenolics, anthocyanins, and terpenoids (Ballaré, 2014). Nonetheless, by carefully adjusting the R/FR ratio, it is possible to enhance both the biomass and the production of valuable bioactive compounds, such as Lancemaside A in *C. lanceolata*.

Various light spectra are known to modulate specific enzymatic activities involved in terpenoid biosynthesis, and this regulation is often influenced by the daily light integral (DLI) (Contreras-Avilés et al., 2024). Terpenoids are high-demand metabolites that have become important targets for understanding plant photomorphogenic responses. In particular, red and blue light act as co-dependent signals that regulate the biosynthesis of various terpenoids via light-induced signaling pathways. In this context, a recent study demonstrated that adjusting red, blue, green, and far-red light ratios in basil cultivation could enhance terpenoid accumulation (Semenova et al., 2022), supporting the idea that light quality manipulation can effectively control the production of bioactive compounds.

Moreover, the addition of FR light not only increased the Lancemaside A content but also enhanced the overall biomass of *C. lanceolata*, contributing to a greater total amount of Lancemaside A per plant. This demonstrates that FR light is an effective strategy for boosting both growth and secondary metabolite production in *C. lanceolata*.

However, while this study presents interesting findings regarding the macroscopic and biochemical phenotypic responses of *C. lanceolata* to different R/FR ratios, the underlying regulatory mechanisms remain unclear. To further elucidate how FR light affects secondary metabolite biosynthesis at the molecular level, future studies should incorporate transcriptomic and metabolomic analyses to provide deeper insights into the underlying regulatory mechanisms. These approaches would help clarify whether JA signaling is directly involved in FR-mediated metabolic regulation, and identify key pathways that may be activated or suppressed under different light conditions.

### 3.4 Linear impact of blue, green, R and FR light ratios on plant growth, Lancemaside A, and secondary metabolites

**Figure 7:**
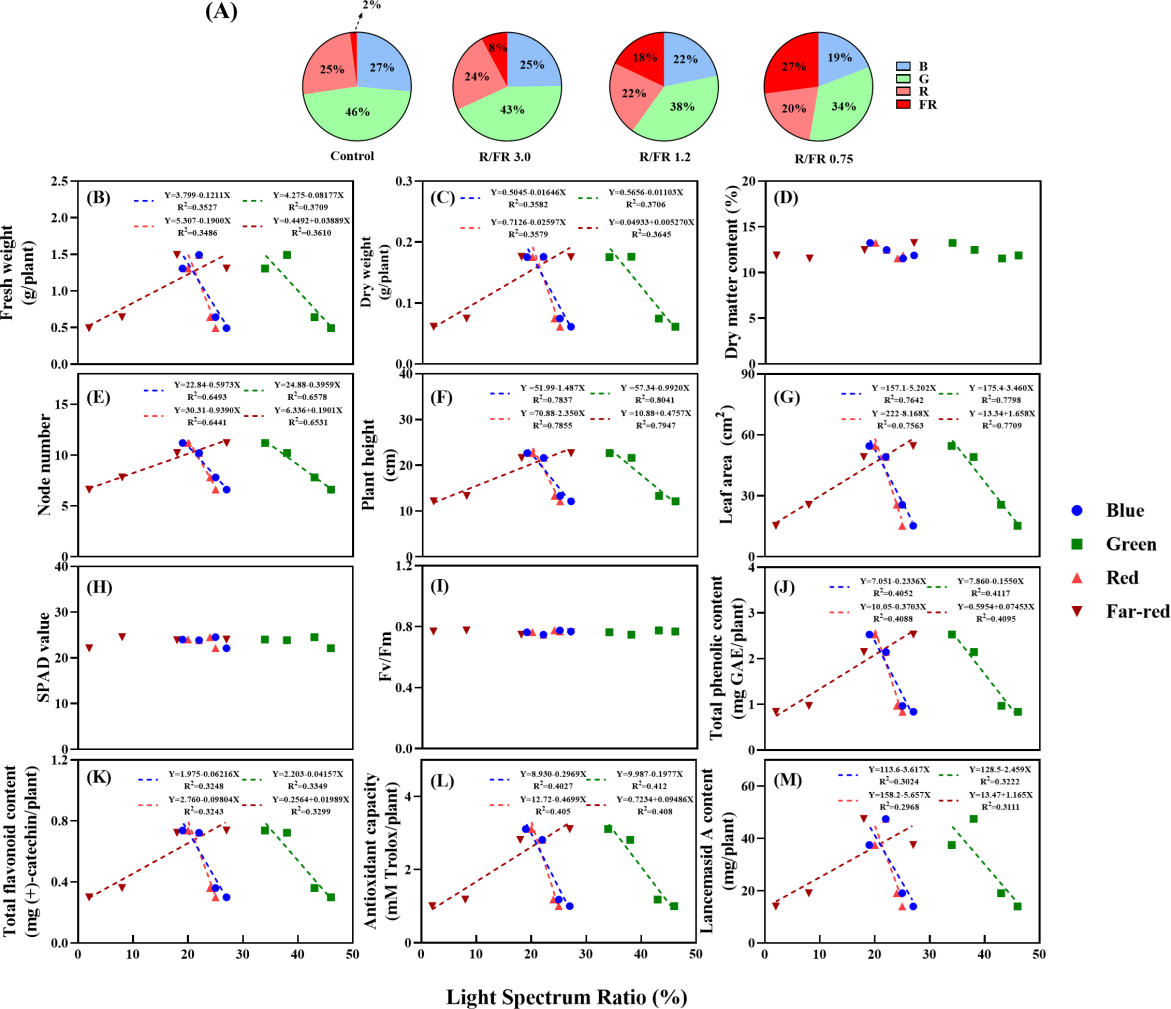
Simple linear regression showing the variation in growth parameters and secondary metabolites based on the light spectrum ratio (%) of various blue, green, red, and far-red. Fresh weight **(B)**, dry weight **(C)**, dry matter content **(D)**, node number **(E)**, plant height **(F)**, leaf area **(G)**, SPAD value **(H)**, Fv/Fm **(I)**, total phenolic content **(J)**, flavonoid content **(K)**, antioxidant capacity **(L)**, and Lancemaside A content **(M)** under various red, blue green and far-red light ratios **(A)**. Each dotted linear regression line shows that the effect of the light spectrum ratio percentage is significant at *p*< 0.05. R^2^ represents the corresponding determination coefficient.

When the total PPFD was kept constant and only the R/FR ratio was adjusted, the composition of blue, green, and R light changed accordingly. As the R/FR ratio decreased, the FR light increased, and the proportion of R light relative to FR light generally decreased. This adjustment can unintentionally reduce the proportion of blue and green light in the entire spectrum, even while maintaining a constant PPFD level. Consequently, the growth parameters and secondary metabolite patterns were analyzed based on the ratios of blue, green, R, and FR light (**Figure 7**).

The DMC, SPAD, and Fv/Fm values (**Figures 7D, H, I**) did not significantly differ with increasing or decreasing ratios of blue, green, R, or FR light. These results suggest that DMC, SPAD, and Fv/Fm were not affected by changes in the light spectrum ratios. As the proportion of FR light increased, the FW, DW, node number, plant height, leaf area, total phenolic content, total flavonoid content, antioxidant capacity, and Lancemaside A content exhibited positive linear relationships. Conversely, a negative linear trend was observed with increasing blue, green, and R ratios (**Figures 7B, C, E–G, J–M**).

The observed decreases in biomass with increasing blue light ratios are consistent with those reported in previous studies. For example, studies have shown that an increase in blue light can reduce growth rates and lead to smaller leaf sizes and lengths, potentially owing to the inhibitory effects of blue light on elongation and expansion (Hogewoning et al., 2010). In addition, green light interferes with blue light signaling pathways, further diminishing growth and yield (Bouly et al., 2007). Similarly, in lettuce, increasing green light ratios were linked to reduced biomass, which was attributed to competitive interactions between green and blue light signals (Kang et al., 2016). When growing leafy green vegetables indoors, appropriate proportions of far-red light can increase biomass accumulation; however, especially under high green light conditions, there may be trade-offs in crop quality (Kelly and Runkle, 2024).

However, higher FR light proportions typically induce shade-avoidance responses, promoting plant growth and expansion by increasing leaf size and stem elongation (Pierik et al., 2004; Keuskamp et al., 2011). Shade-avoidance responses are triggered by increased FR and lower blue light levels, signaling dense canopy conditions (Vandenbussche et al., 2005). These responses explain the observed increases in node number, plant height, and leaf area as the FR light ratio increased, which contributed to enhanced biomass and secondary metabolite content. Blue light, although beneficial for branching and compact morphology, consumes more energy and generally suppresses elongation. In contrast, red light, with its longer wavelength, promotes stem and leaf growth, generating higher overall biomass. Therefore, finding the right balance among these light spectra is crucial for maximizing plant growth and quality (McKay et al., 2025).

Despite these growth-promoting effects, an excessive proportion of green light did not significantly enhance the growth parameters. This result aligns with findings indicating that adding green light alone does not effectively trigger shade-avoidance responses without sufficient FR light supplementation (Kang et al., 2016). Therefore, the interaction between different spectral compositions is essential for achieving optimal growth and metabolite synthesis under specific environmental conditions. Failure to properly balance these interactions may lead to impaired growth and reduced productivity.

Traditionally, shade-avoidance responses have been primarily attributed to R/FR ratios. However, accumulating evidence indicates that plants also adjust their growth in response to reductions in blue light intensity (Pierik et al., 2004; Millenaar et al., 2005). These findings suggest a complex regulatory network in which multiple light signals, including blue, green, and FR light, play significant roles in shaping plant morphology and productivity.

Future research is needed to further investigate how the dynamic interactions among these light spectra influence key regulatory pathways, which could help optimize spectral compositions for improved plant productivity and bioactive compound accumulation.

### 3.5 Optimizing daily light integral, light use efficiency, and energy use efficiency through FR light supplementation

**Figure 8:**
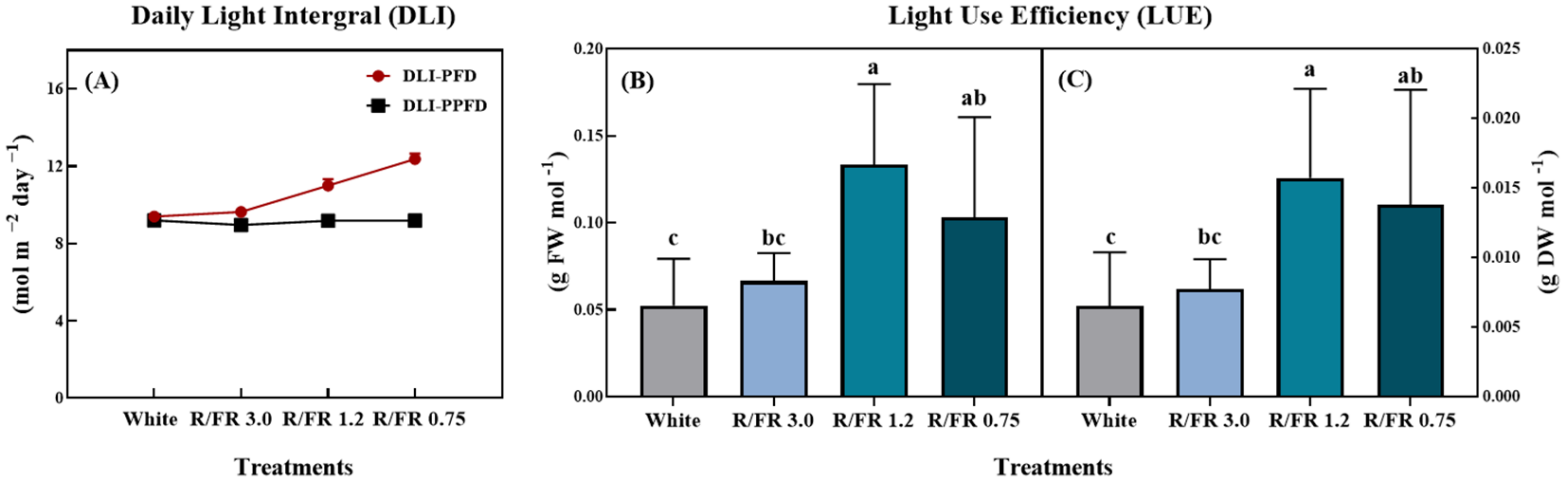
Effect of the red/far-red (R/FR) ratio on the daily light integral **(A)** and light use efficiency fresh **(B)** and dry weight **(C)** of *C*. *lanceolata* spouts. The vertical bars represent the standard deviations (n = 10).

As shown in **Figure 8**, the DLI and LUE of *C. lanceolata* sprouts were significantly affected by the R/FR ratio treatments. The DLI calculated from the PFD and PPFD (**Figure 8A**) showed an increasing trend across the treatments, with the highest value observed in the R/FR 0.75 treatment. In contrast, the DLI-PPFD values remained relatively stable in all treatments, and the changes in DLI-PFD were mainly caused by the addition of FR light.

The FW (**Figure 8B**) and DW (**Figure 8C**), which indicate the LUE, also differed significantly among treatments. From a physiological perspective, it is insightful to consider LUE as the ratio of leaf dry weight (LDW) to the total photon flux provided to the canopy (Jin et al., 2020).

Both the LUE-FW and LUE-DW were highest in the R/FR 1.2 treatment, followed closely by the R/FR 0.75 treatment, with both treatments showing significantly higher values than the white control and R/FR 3.0 treatment. These results suggest that the increased FR component in the 1.2 R/FR ratio enhanced the efficiency of *C. lanceolata* utilizing light for biomass production. This may be due to increased resource allocation for growth, resulting in increased FW and DW yields.

In this experiment, differences in DLI observed across treatments were primarily due to the addition of FR light, which increased the PFD while maintaining the same baseline PPFD. As PPFD measures only photosynthetically active radiation (PAR) ranging from 400–700 nm, it remained consistent across treatments. However, with the addition of FR light, which falls outside the traditional PAR range (typically approximately 700–750 nm), the PFD values increased, thereby increasing the overall DLI. This setup allowed a comparison of how FR light affects DLI without altering the base PAR intensity, thus isolating the influence of FR light on light use efficiency and plant growth responses.

**Figure 9:**
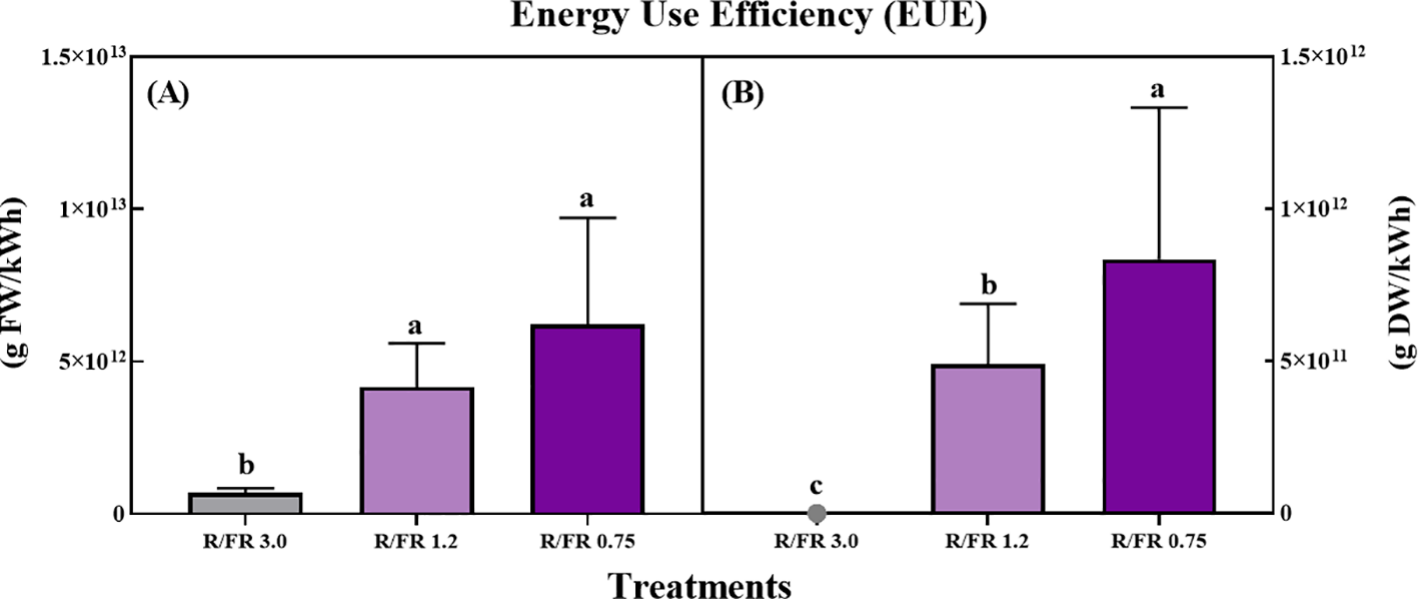
Effect of red/far-red (R/FR) ratio on the energy use efficiency (EUE) for fresh weight **(A)** and dry weight **(B)** production in *C*. *lanceolata* sprouts across three treatments. The white control was excluded as no far-red light was applied. In the dry weight graph **(B)**, the EUE value for the R/FR 3.0 treatment was too small to display visibly. Vertical bars represent the standard deviations (n = 10). The EUE values were calculated based on the energy consumed during the 10-day far-red treatment period, with each treatment consuming a total of 7.2 kWh due to the continuous operation of far-red LEDs for 12 h per day.

For the analysis of environmental and economic sustainability, it is reasonable to express LUE in grams of FW and DW per kilowatt used by the system, that is, the lighting EUE. The results presented in **Figure 9** demonstrate that the EUE for both FW (A) and DW (B) of *C. lanceolata* sprouts was significantly affected by the R/FR ratio treatments.

For FW (**Figure 9A**), EUE gradually increased as the R/FR ratio increased, with the highest EUE observed in the R/FR 0.75 treatment, followed by R/FR 1.2, and the lowest observed in R/FR 3.0.

This indicates that a higher proportion of FR light enhances the energy efficiency of fresh biomass production. Similarly, for DW (**Figure 9B**), EUE was significantly higher in R/FR 0.75, followed by R/FR 1.2, whereas the EUE value for the R/FR 3.0 treatment was too small to be displayed visibly on the graph. These findings suggest that FR light supplementation is effective in increasing EUE, particularly at lower R/FR ratios, where biomass accumulation is more efficiently supported.

This finding is related to the ability of moderate exposure to far-red light to stimulate photosynthetic efficiency and biomass accumulation without significantly increasing energy consumption. Although far-red photons are less effective at directly inducing photosynthesis those within the PAR range (400–700 nm), far-red light can induce an Emerson enhancement effect, where photosynthetic rates are enhanced due to the synergistic interaction between far-red and red light (Zhen et al., 2021). This synergistic effect may have contributed to the observed increase in biomass accumulation relative to the energy input at certain R/FR ratios.

FR light may also affect plant morphology by increasing the efficiency of light interception by the canopy structure. FR light supplementation has been shown to promote stem elongation and leaf expansion, which may improve light capture, particularly in dense plant canopies. This improved light capture efficiency means that plants can utilize the available light more effectively, thus increasing biomass production per unit of energy (Meng and Runkle, 2019). This aligns with findings suggesting that FR supplementation can promote canopy architecture that is favorable for light capture, ultimately supporting higher LUE values, as seen in the R/FR 1.2 and 0.75 treatments (Carotti et al., 2024).

Thus, increasing the number of plants produced per unit of electricity by adding FR light can be achieved by controlling the phytochrome effect (Martin and Molin, 2019).

### 3.6 Correlation analysis of growth characteristics and metabolite content under different R/FR ratios

**Figure 10:**
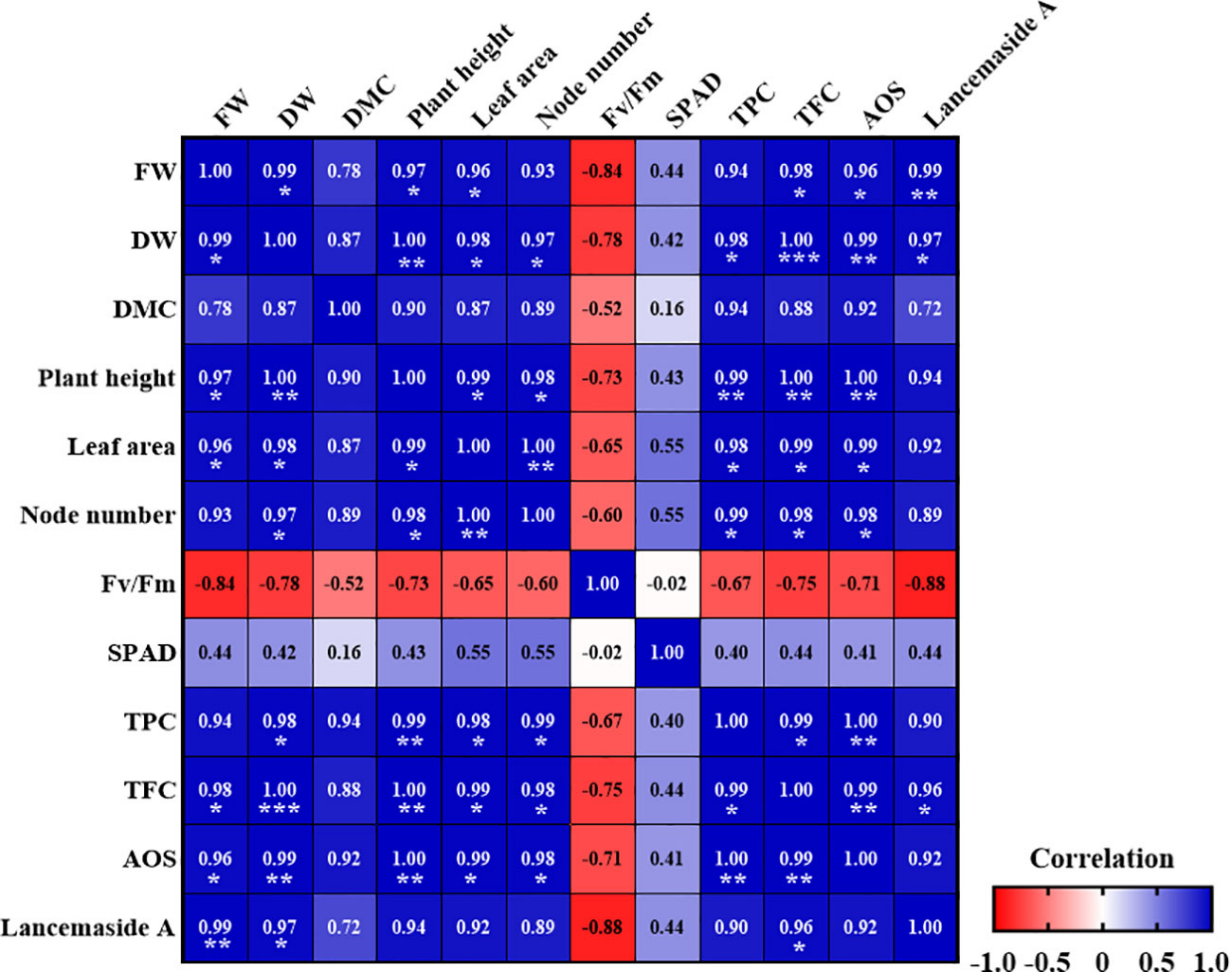
Pearson correlation matrix average growth parameters and physiological characteristics in *Codonopsis lanceolata* sprouts under different red/far-red (R/FR) ratios. Values are Pearson correlation coefficients, which are used to construct the heat map. FW, Fresh weight; DW, Dry weight; DMC, Dry matter content; TPC, Total phenolic content; TFC, Total flavonoid content, and AOS, Antioxidant capacity. *Significant at *p* < 0.05, **Significant at *p* < 0.01, ***Significant at *p* < 0.001. The heatmap shows negative and positive correlations.

We performed correlation analyses to determine the effects of the R/FR ratios on the growth and physiological parameters of *C. lanceolata* sprouts (**Figure 10**). We observed significant correlations among the 12 growth and physiological parameters in *C. lanceolata* under different R/FR ratios. The correlation coefficients ranged from -0.88 to 1.0, indicating both strong positive and negative associations. The FW and DW were strongly positively correlated with plant height, leaf area, total phenolic content (TPC), total flavonoid content (TFC), and antioxidant capacity (AOS) (*p<* 0.05, *p*< 0.01). This indicates that as the biomass increased, the levels of phenolic and flavonoid compounds also increased, thereby enhancing the overall antioxidant capacity of the plant. These findings suggest a close relationship between the accumulation of these secondary metabolites and the antioxidant properties of *C. lanceolata* sprouts. Conversely, Fv/Fm exhibited a strong negative correlation with FW, DW, and other growth parameters, indicating a trade-off between photosynthetic efficiency and biomass accumulation.

Additionally, the Lancemaside A content was positively correlated with the TFC (*p<* 0.05), suggesting that the accumulation of secondary metabolites was associated with higher flavonoid content. The heat map illustrates the distinct relationships between these parameters, where positive correlations are shown in blue and negative correlations are shown in red.

The positive correlations observed between the FW and DW with the TPC and TFC contents suggest that higher biomass accumulation is associated with elevated levels of secondary metabolites. This relationship indicates that as plants grow larger, they may allocate more resources to the synthesis of secondary metabolites, thereby enhancing their phenolic and flavonoid contents (Bae et al., 2017). Similarly, in *P. vulgaris*, the FR light treatment was found to increase the TPC, rutin content, rosmarinic acid content, DPPH radical scavenging activity, and reducing power, indicating concurrent enhancement (Chen et al., 2023). Such a trend could be advantageous for optimizing cultivation practices to simultaneously improve both the yield and phytochemical quality of *C. lanceolata*.

Although the ANOVA results showed no significant differences in the Fv/Fm across treatments, the Pearson correlation analysis revealed a weak negative relationship between Fv/Fm and certain growth parameters. This suggests that although photosynthetic efficiency, as measured by Fv/Fm, remained generally stable across all treatments, small changes in Fv/Fm values were observed in relation to increased growth parameters, such as FW and DW. These slight variations in Fv/Fm might be linked to the plants allocating more resources toward growth, leading to minor trade-offs in photosynthetic efficiency. However, these subtle trends were not sufficient to produce statistically significant changes in Fv/Fm between treatments.

Furthermore, the significant positive correlations between Lancemaside A content and TPC, TFC, and antioxidant capacity indicate a potential link between the synthesis of this compound and the antioxidant properties of the plant. This suggests that the production of Lancemaside A may be part of a coordinated metabolic response that enhances antioxidant defense in *C. lanceolata*. Ryu et al. (1997) reported a strong positive correlation between polyphenol contents and antioxidant activity, suggesting that these compounds significantly contribute to the high antioxidant capacity observed. Other studies on *C. lanceolata* roots have demonstrated superior anti-inflammatory activity, which is likely closely related to the higher content of functional compounds, including Lancemaside A (Kim et al., 2022). The interplay among plant growth, secondary metabolite content, and antioxidant capacity underscores the complex regulatory networks governing plant development and stress responses at varying R/FR ratios. These findings suggest that optimizing light conditions, particularly the R/FR ratio, can be an effective strategy for enhancing both biomass yields and the accumulation of bioactive compounds, thereby improving the functional and economic value of *C. lanceolata* cultivation.

## 4 Conclusion

Here, we developed a vertical cultivation system for *C. lanceolata* sprouts and investigated how variations in the R/FR ratio influence their growth and bioactive compound accumulation. This study provides a systematic approach to optimizing light spectra in controlled environments, offering practical insights into the use of FR supplementation to enhance biomass production and secondary metabolite accumulation. Our findings demonstrate that FR supplementation significantly increased biomass, leaf area, and node number, while also elevating total phenolic, flavonoid, and antioxidant contents, as well as Lancemaside A accumulation. These results establish a foundational dataset for optimizing light quality in the cultivation of *C. lanceolata* and similar medicinal crops, supporting the potential for improved functional crop production in plant factories.

However, plant growth and secondary metabolite accumulation can be influenced by multiple factors beyond the R/FR ratio, including developmental stage (e.g., age, leaf thickness) and environmental conditions in vertical farming systems. Further studies considering these factors are necessary to enhance the validity and generalizability of these findings.

Additionally, while this study provides valuable insights into the physiological and biochemical responses of *C. lanceolata* to FR light, the underlying regulatory mechanisms remain unclear. Future research incorporating transcriptomic and metabolomic analyses is needed to elucidate how FR light modulates secondary metabolite biosynthesis at the molecular level, which could aid in developing targeted light management strategies for enhanced *C. lanceolata* production.

## Data Availability

The raw data supporting the conclusions of this article will be made available by the authors, without undue reservation.
